# Interaction between Facial Expression and Color in Modulating ERP P3

**DOI:** 10.1523/ENEURO.0419-24.2024

**Published:** 2025-01-08

**Authors:** Yuya Hasegawa, Hideki Tamura, Shigeki Nakauchi, Tetsuto Minami

**Affiliations:** Department of Computer Science and Engineering, Toyohashi University of Technology, Toyohashi 441-8580, Japan

**Keywords:** attention, EEG, ERP, facial color, facial expression, P300

## Abstract

The relationships between facial expression and color affect human cognition functions such as perception and memory. However, whether these relationships influence selective attention and brain activity contributed to selective attention remains unclear. For example, reddish angry faces increase emotion intensity, but it is unclear whether brain activity and selective attention are similarly enhanced. To investigate these questions, we examined whether event-related potentials for faces vary depending on facial expression and color by recording electroencephalography (EEG) data. We conducted an oddball task using stimuli that combined facial expressions (angry, neutral) and facial colors (original, red, green). The participants counted the number of times a rarely appearing target face stimulus appeared among the standard face stimuli. The results indicated that the difference in P3 amplitudes for the target and standard faces depended on the combinations of facial expressions and facial colors; the P3 for red angry faces were greater than those for red neutral faces. Additionally, facial expression or facial color had no significant main effect or interaction effect on P1 amplitudes for the target, and facial expression had significant main effects only on the N170 amplitude. These findings suggest that the interaction between facial expression and color modulates the P3 associated with selective attention. Moreover, the response enhancement resulting from this interaction appears to occur at a cognitive processing stage that follows the processing stage associated with facial color or expression alone. Our results support the idea that red color increases the human response to anger from an EEG perspective.

## Significance Statement

Whether the relationships between facial expression and color influence selective attention and its brain activity remains unclear. Using an oddball task and recording EEGs, we showed that the ERPs reflecting selective attention are modulated by the interaction between facial expression and color, although this interaction was not found at earlier ERP stages. These findings suggest that the intensity of ERP associated with selective attention to facial expressions is influenced more by the interaction between facial expression and facial color than by facial expression or facial color alone and that the interaction occurs as a higher-order processing stage than facial expression or color recognition. Our results provide EEG evidence supporting the idea that red color increases the human response to anger.

## Introduction

Facial color affects the judgment of facial expressions, with reddish faces easily judged as an angry face and perceived as having a greater emotional intensity of anger (Nakajima et al., 2017; Minami et al., 2018; Peromaa and Olkkonen, 2019; Thorstenson et al., 2021; Kato et al., 2022). Additionally, facial color has been shown to influence perceived social characteristics such as friendliness, aggression, and health (Thorstenson and Pazda, 2021). Moreover, the effects of the relationship between facial expression and facial color are similar not only for real faces but also for emoticons, facial models, and implicit faces; this relationship is also known to change with background color, suggesting that specific colors enhance emotion perception (Liao et al., 2018; Minami et al., 2018; Qin, 2021; Thorstenson et al., 2022; Nguyen et al., 2023). Recent studies have focused on the relationship between facial expression and facial color memory and suggested that facial expression biases facial color memory (Thorstenson et al., 2021; Hasegawa et al., 2024). Thus, the relationship between facial expression and facial color, especially the relationship between anger and red, affects cognitive function, such as human judgment and memory. Red angry faces increase the perception of anger (Nakajima et al., 2017), whereas angry faces increase selective attention (Öhman et al., 2001; van Honk et al., 2001; Fox et al., 2002; Mogg et al., 2004). In the relationship between facial expression and selective attention, angry and fearful faces are known to bias visual attention, and it is believed that perceived threats may be among the factors that capture human attention (Öhman et al., 2001; Fox et al., 2002; Mathews et al., 2003). However, it is still unclear whether the relationship between facial expression and color affects selective attention and its brain activity, which are also modulated by facial expression. Therefore, we hypothesize that red angry faces, which are perceived as expressing more anger, also increase selective attention, and this hypothesis was tested via an approach based on electroencephalography (EEG).

The event-related potential (ERP) P3 is a measure for comparing EEGs associated with selective attention. P3 is an ERP component that occurs ∼300–500 ms after a presented stimulus at the parietal lobe as the third positive deflection; P3 is also known to reflect selective attention. The oddball paradigm is a task that induces a large P3 amplitude. In the oddball task, participants count how often a low-frequency or specified stimulus appears among stimuli at other frequencies. Low-frequency stimuli elicit large P3 amplitudes during the oddball task. With respect to the relationship between facial expressions and P3 amplitudes, previous studies have shown that attention to angry or fearful faces increases the P3 amplitude (Rossignol et al., 2005; Kiss and Eimer, 2008; Chai et al., 2012; Lin et al., 2020). Thus, angry faces affect the P3 amplitude and selective attention. However, it is still unclear whether the addition of red color, which is strongly associated with anger, increases P3 amplitudes and selective attention.

Therefore, this study aimed to clarify the effect of the relationship between facial expression and facial color on the P3, which reflects selective attention. The P3 amplitude varies in magnitude depending on the intensity of selective attention to the target. Therefore, we hypothesized that when the relationship between anger and red causes strong selective attention, an interaction effect of facial expression and facial color is exerted on P3 rather than a color effect alone. For example, the P3 amplitude for red angry faces is larger than that for red neutral faces, and this trend is more pronounced than when angry faces with normal facial color are compared with neutral faces with normal facial color. In this study, we recorded participants’ electroencephalography (EEG) during an oddball task to investigate the effects of facial expression and facial color on EEG, which reflects selective attention. Then, we compared the P3 amplitudes to estimate how differences in facial expression and facial color influence selective attention. Additionally, the amplitudes of P1 and N170 were also examined in this study. P1 is an ERP component that occurs ∼100 ms after a presented stimulus in the occipital area and reflects the initial visual processing (Hillyard and Anllo-Vento, 1998; Colombatto and McCarthy, 2017). Previous studies reported that P1 modulation was unaffected by facial expression factors (Schindler and Bublatzky, 2020). N170 is a negative ERP component that is observed ∼170 ms after stimulus presentation in the left and right posterior temporal regions and is sensitive to facial stimuli (Bentin et al., 1996). N70 is modulated by facial expression and facial color (Minami et al., 2011; Nakajima et al., 2012; Hinojosa et al., 2015). In this study, we also investigated whether the relationship between facial expression and color is observed from ERP stages prior to the P3, such as P1 and N170.

## Materials and Methods

### Participants

Twenty Japanese students (5 women and 15 men; mean age, 22.50 ± 1.00 years) at Toyohashi University of Technology participated in the experiment. The sample size was calculated using PANGEA (Westfall et al., 2014) with an effect size of d=0.4, power=0.8, and α=0.05, and we found that 19 participants were needed. Assuming a possibility of data rejection due to any EEG artifacts, we recruited 20 participants. Before joining the experiment, the participants were provided with an introduction to the experiment, excluding the study's hypothesis, and gave informed consent. All participants had normal color vision, as verified by the Ishihara Color Vision Test Chart II Concise Version 14 Plate (the Public Interest Incorporated Foundation Isshinkai, Handaya). This experiment was conducted with the approval of the Ethics Committee for Human Research at Toyohashi University of Technology and adhered strictly to the approved guidelines of the committee and the Declaration of Helsinki. This study was not preregistered.

### Stimuli

**Figure 1.**
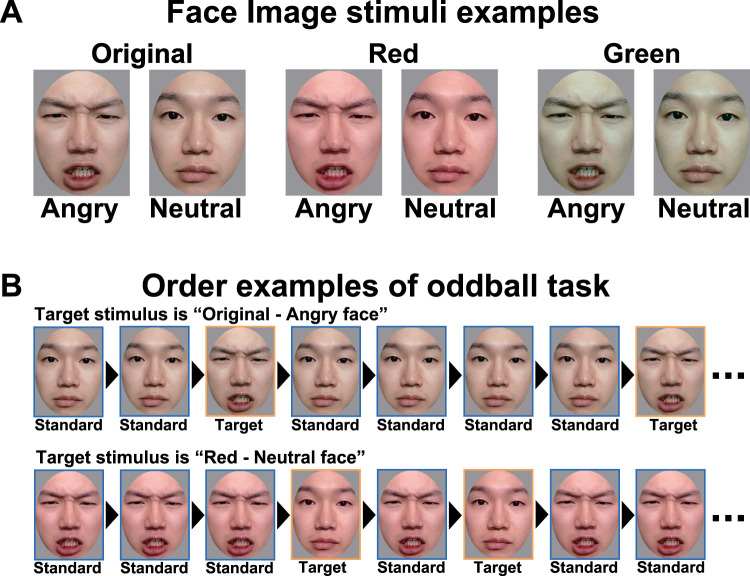
***A***, Examples of the image stimuli for each condition. There were two facial expression conditions, angry and neutral, and three facial color conditions, original (no manipulation), red (a* + 12 units), and green (a* − 12 units). Two face models (1 woman and 1 man) were used for each of the six conditions (2 facial expression × 2 facial color). ***B***, Examples of presentation order for the oddball task. Standard stimuli are presented at high frequency, and target stimuli are presented at low frequency. The faces in the figure are from one of the authors (Y.H.) and were not used in the experiment.

The facial stimuli (Fig. 1) were angry and neutral faces of two Japanese individuals (one woman and one man) obtained from the ATR Facial Expression Image Database (ATR-Promotions; https://www.atr-p.com/products/face-db.html). The hair, ears, and necks in the images were removed via Photoshop (Adobe Systems), with the edited image presenting an oval shape. All the images were adjusted to maintain an average image luminance of $16.9\;\;\mathrm{cd}/m^{2}$ via SHINE_color, a MATLAB 2021a toolbox (Willenbockel et al., 2010; Ben, 2021). Based on an experiment by Nakajima et al. (2017), we created colored facial stimuli by manipulating the a* (red–green) value in CIE L*a*b* (Nakajima et al., 2017). There were three facial color conditions: original (no manipulation), red (a* + 12 units), and green (a* − 12 units). Twelve image stimuli (2 individuals × 2 facial expressions × 3 facial colors) were prepared. The image dimensions were $4.2^{\circ }\times 5.5^{\circ }$. The mean and standard deviation of the CIE L*a*b* values for the original faces were L*=47.60±0.06, a*=7.61±0.09, and b*=22.41±0.06. The background color was always gray $\left(Y=17.09\;\;\mathrm{cd}/m^{2}\right)$.

### Apparatus

The experiment was conducted in a dark magnetically shielded room. The stimuli were presented on a monitor (VIEPixx/EEG, VPixx Technologies; resolution: 1,920 × 1,080; frame rate 120 Hz). The white point of the monitor was $[x,y]=[0.30,\;0.33],\;Y=91.23\;\;\mathrm{cd}/m^{2}$. The participants were seated and performed the task while keeping their heads on a chin rest positioned 60 cm from the display. Psychtoolbox 3.0.17 served as the experimental control software (Brainard, 1997; Pelli, 1997; Kleiner et al., 2007). EEG data were acquired via 64 channels of electrodes and six channels of external sensors at a sampling frequency of 512 Hz via BioSemi ActiveTwo and recorded via the ActiveTwo System.

### Procedure

**Figure 2.**
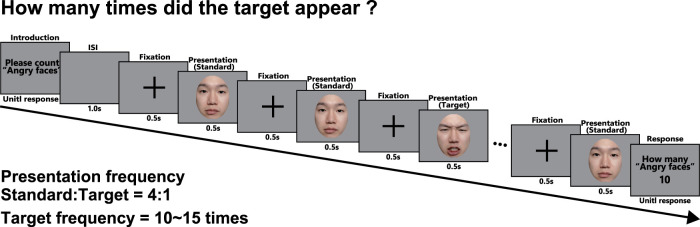
Procedure of the experiment when the target stimuli are angry faces. In the repetition presentation phase, the stimuli were presented in a random order with a frequency of Target: Standard = 1: 4. After the presentation phase, the participant recorded the number of times the specified facial stimulus (target stimulus) appeared in the instruction phase using a numeric keypad. The ratio among the number of text screens, fixation crosses, and stimuli depicted in this figure differs from the actual ratio.

In the experiment, we used six image stimulus pairs. Three pairs were prepared for each face stimulus (two persons): the original color angry face and original color neutral face, the red angry face and red neutral face, and the green angry face and green neutral face. An oddball task was performed with the high-frequency stimulus as the standard stimulus and the low-frequency stimulus as the target stimulus in these pairs. In each trial, when the target stimulus was a red angry face, the standard stimulus was a red neutral face, and when the target stimulus was a red neutral face, the standard stimulus was a red angry face. Therefore, a participant performed 12 trials (6 pairs × 2 targets) of the oddball task in total. Standard and target stimuli were presented in a random order during each trial. The frequency of standard and target stimuli was always Standard:Target=1:4, and the target stimulus was presented 10–15 times. The participants were asked to count the number of times the target stimulus appeared. Figure 2 shows a summary of the experimental procedure. First, a task description was presented until the participant pressed the enter key. After a 1.0 s interstimulus interval, the fixation points and facial expression stimuli (standard or target stimulus) were presented repeatedly at 0.5 s intervals. After the end of the presentation, the participant recorded the number of times the specified facial stimulus (target stimulus) appeared via a numeric keypad. The experiment was conducted in two blocks, one for counting angry faces and one for counting neutral faces (6 trials per block), and the order of the blocks differed among the participants. The order of the six pairs (2 persons × 3 facial color) presented within a block was random. The participants could take breaks between trials and blocks. The participants wore EEG equipment throughout the experiment, and their EEGs were recorded during the task. Additionally, to reduce motion noise, the participants were instructed to avoid counting with their fingers or voices.

### Preprocessing of EEG data

For preprocessing, the EEG data were downsampled to 200 Hz, and a high-pass filter (1 Hz) and the function “cleanLineNoise” in EEGLAB were applied to eliminate line noise such as white noise, power supply noise (60 Hz), and its harmonic frequencies (120, 180, and 240 Hz). The significance cutoff level was p=0.01. Additionally, electrodes that did not measure the data well were removed via the function “clean_rawdata” in the EEGLAB tool (unchanged interval: 5 s, correlation with surrounding electrodes: <0.85, far from average: 4 times the standard deviation, removal via the ASR algorithm). The electrode data excluded by “clean_rawdata” were subsequently interpolated via the spherical spline interpolation method via the use of peripheral electrode data. Moreover, artifact removal was performed by eliminating ocular components via adaptive mixture independent component analysis (AMICA) and ICLabel (Leutheuser et al., 2013; Pion-Tonachini et al., 2019). Finally, we set the time of stimulus presentation as 0ms and extracted the EEG data at −100 to 1,000 ms. One participant was excluded from the analysis because this preprocessing excluded all EEG data of target stimuli for one condition.

### Statistical analysis

#### P3

First, we extracted the channel-averaged EEG at Cz, CPz, CP1, CP2, and Pz from the preprocessed EEG. The baseline EEG was the average EEG of −100 to 0 ms. Next, the mean amplitudes at 300–500 ms after the presentation of the standard and target stimuli were calculated for each trial. In this experiment, the time windows and channels used were predefined during the experimental design phase based on previous research (Polich, 2007; Hinojosa et al., 2015; Che et al., 2024). Then, the mean amplitude of the standard stimulus was subtracted from the mean amplitude of the target stimulus during the same trial, and we calculated the average for each of the facial expression (anger, neutral) and facial color (original, red, green) conditions (for a total of six conditions). Quartile range exclusion was subsequently performed for each condition to exclude outliers. Thus, the data of one participant with an outlier outside the interquartile range were excluded. Therefore, the final statistical analysis included 18 participants.

We first conducted the Shapiro–Wilk test to confirm that the data were normally distributed. If the data were normal, we performed a repeated-measures two-way analysis of variance using R; otherwise, we performed a nonparametric test with nparLD, an R software package (Noguchi et al., 2012). ANOVA-type statistics (ATS) were calculated via nonparametric tests. Moreover, the *p* values were subjected to post hoc correction via the Holm method.

#### N170 (left, right)

We extracted the channel-averaged EEG at five channels near the temporal area (left: TP7, P5, P7, P9, PO7; right: TP8, P6 P8, P10, PO8) from the preprocessed EEG. The baseline time window was the same as that for the P3 amplitude. Then, the peak amplitudes at 150–200 ms after the presentation of the target stimuli were calculated for each trial, and we averaged them for each condition via the same method as that used for the P3. Afterward, the same statistical analysis as that used for P3 was performed on the left and right peak amplitudes. We used the same participant data as those used in the statistical analysis of the P3.

#### P1

We extracted the channel-averaged EEG at Iz, Oz, O1, O2, and POz from the preprocessed EEG. The baseline time window was the same as that for the P3 and N170 amplitudes. The P1 amplitude was calculated via the same procedure used for N170, except that the time window of the peak amplitude was 80–120 ms. Afterward, the same statistical analysis as that used for P3 was performed on the left and right peak amplitudes. We used the same participant data as those used in the statistical analysis of the P3.

## Results

### P3

**Figure 3.**
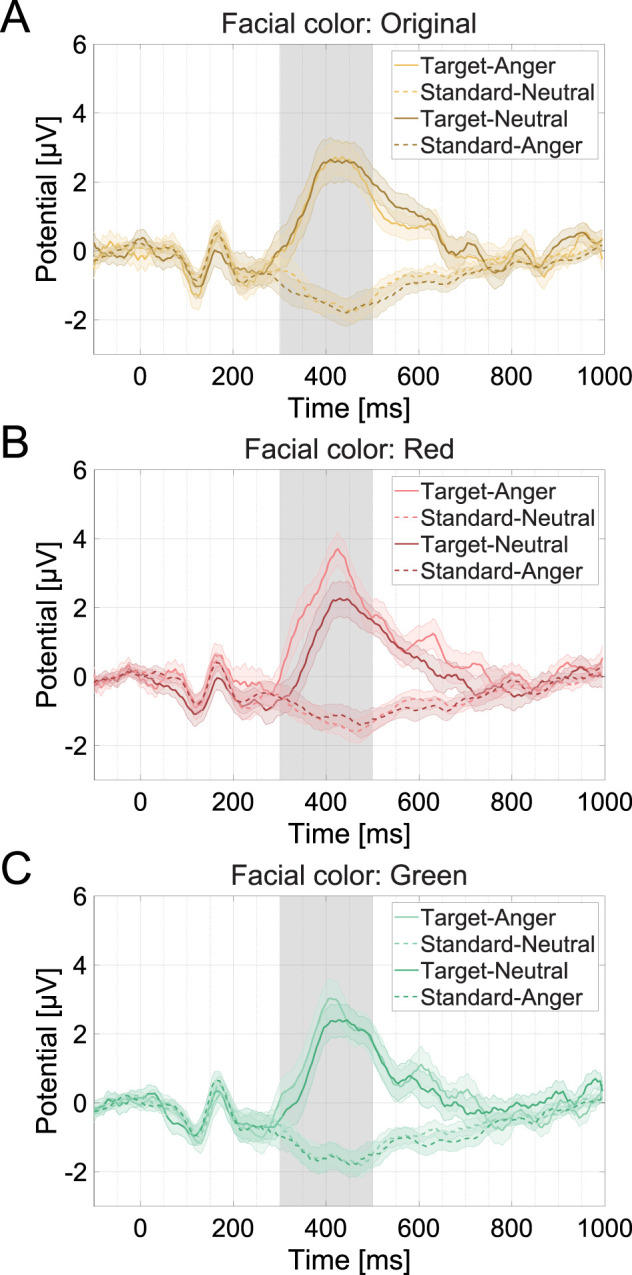
Channel-averaged EEG waves of the mean for each condition (***A***, original; ***B***, red; ***C***, green). The bands covering the unbroken curve and the dashed curve represent the standard error of the mean. The gray bands at 300–500 ms are the time windows of P3 analysis. The data were smoothed for plotting and were not used in the analysis.

**Figure 4.**
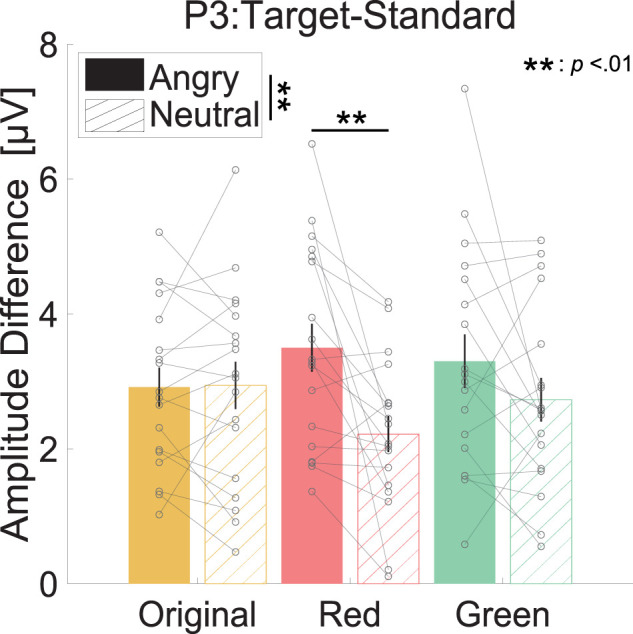
Mean of the difference in P3 amplitude [μV] for target and standard stimuli. Each gray point represents individual data. The color of each bar and the label on the horizontal axis indicate facial conditions. The filled and hatched bars indicate that target stimuli were angry faces and neutral faces, respectively. Error bars show the standard error of the mean.

Figure 3 shows the average wave for the target and standard stimuli for each condition. Figure 4 shows the mean P3 amplitude for each facial expression and facial color condition. We found a significant main effect of facial expression $\left(F_{\left(1,17\right)}=10.089,\;p<0.01,\;\eta_{p}^{2}=0.372\right)$ and a significant interaction effect between facial expression and facial color $\left(F_{\left(1.97,33.48\right)}=3.747,\;p<0.05,\;\eta_{p}^{2}=0.181\right)$. The post hoc results revealed that the P3 amplitude for angry faces was greater than that for neutral faces $\left(t_{\left(17\right)}=3.176,\;p<0.01,\;{\mathrm{Cohen}}^{\prime }s\;d=0.749\right)$, and the P3 amplitude for red angry faces was greater than that for red neutral faces $\left(t_{\left(17\right)}=3.382,\;p<0.01,\;{\mathrm{Cohen}}^{\prime }s\;d=0.797\right)$. The other statistical analysis results for P3 are shown in Tables 1–4.

**Table 1.** Summary of two-way repeated-measures ANOVA results for P3

Effect	F	$df^{\mathrm{GG}}$	${\mathrm{df}}_{\mathrm{res}}^{\mathrm{GG}}$	p	$\eta_{p}^{2}$
Expression	10.09	1	17	0.006	0.372
Color	0.22	1.80	30.53	0.784	0.013
Expression × color	3.75	1.97	33.48	0.035	0.181

**Table 2.** Post hoc comparisons of expression (P3)

Contrast	t	95%CI	df	p	Cohen's *d*
Angry–neutral	3.18	[0.20, 1.01]	17	0.006	0.75

**Table 3.** Summary of main effects analysis for expression × color interaction (P3)

Effect	F	$df^{\mathrm{GG}}$	${\mathrm{df}}_{\mathrm{res}}^{\mathrm{GG}}$	p	$\eta_{p}^{2}$
Expression at original	0.01	1	17	0.914	0.001
Expression at red	11.44	1	17	0.004	0.402
Expression at green	2.28	1	17	0.150	0.118
Color at angry	1.44	1.97	33.42	0.252	0.078
Color at neutral	2.70	1.70	28.98	0.092	0.137

**Table 4.** Post hoc comparisons of expression at red (P3)

Contrast	t	95%CI	df	p	Cohen's *d*
Angry–neutral	3.38	[0.48, 2.08]	17	0.004	0.80

### N170

**Figure 5.**
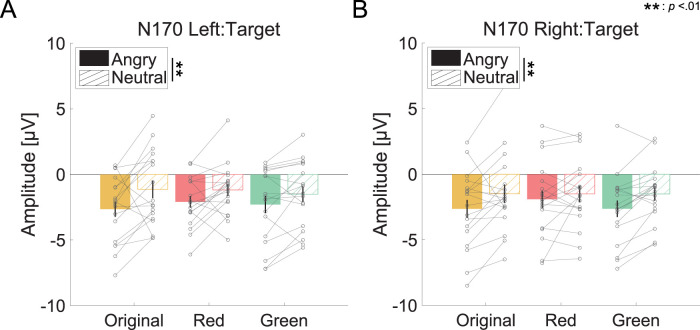
Mean of the N170 amplitude [μV] for target stimuli (***A***, left side; ***B***, right side). The color of each bar and the label on the horizontal axis indicate facial conditions. The filled and hatched bars indicate that target stimuli were angry faces and neutral faces, respectively. Error bars show the standard error of the mean.

Figure 5 shows the mean N170 amplitude for each facial expression and facial color condition. We found a significant main effect of facial expression on the left and right sides (left: $\mathrm{AT}S_{\left(1\right)}=6.940,\;p<0.01$; right: $F_{\left(1,17\right)}=10.100,p<0.01,\eta_{p}^{2}=0.373$). Post hoc tests revealed that the N170 amplitudes for angry faces were greater than those for neutral faces (left: $Z_{\left(17\right)}=-2.896,p<0.01,r=0.683$; right: $t_{\left(17\right)}=-3.178,p<0.01,{\mathrm{Cohen}}^{\prime }s\;d=0.749$). These results are similar to those of previous studies, which revealed larger N170 amplitudes for negative facial expressions than for neutral facial expressions. The other statistical analysis results for N170 are shown in Tables 5–8.

**Table 5.** Summary of nonparametric ANOVA results for N170 left

Effect	$F_{\mathrm{ATS}}$	df	p
Expression	6.94	1	0.008
Color	0.47	1.96	0.621
Expression × color	0.63	1.94	0.529

**Table 6.** Post hoc comparisons of expression (N170 left)

Contrast	Z	df	p	*r*
Angry–neutral	−2.90	17	0.002	0.68

**Table 7.** Summary of two-way repeated-measures ANOVA results for N170 right

Effect	F	$df^{\mathrm{GG}}$	${\mathrm{df}}_{\mathrm{res}}^{\mathrm{GG}}$	p	$\eta_{p}^{2}$
Expression	10.10	1	17	0.006	0.373
Color	0.64	1.68	28.54	0.508	0.036
Expression × color	1.31	1.97	32.18	0.283	0.071

**Table 8.** Post hoc comparisons of expression (N170 right)

Contrast	t	95%CI	df	p	Cohen's *d*
Angry–neutral	−3.18	[−1.49, −0.30]	17	0.006	0.75

### P1

**Figure 6.**
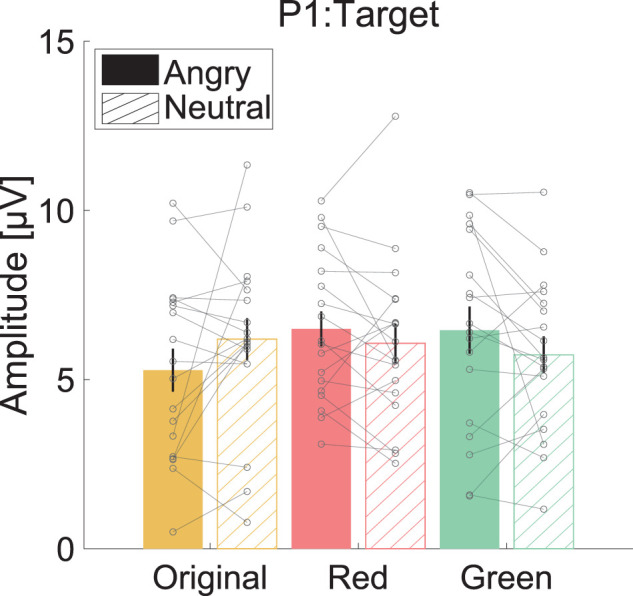
Mean P1 amplitude [μV] for target stimuli. The color of each bar and the label on the horizontal axis indicate facial conditions. The filled and hatched bars indicate that target stimuli were angry faces and neutral faces, respectively. Error bars show the standard error of the mean.

Figure 6 shows the mean P1 amplitude for each facial expression and facial color condition. No significant main effect or interaction effect of facial expression and facial color was exerted (Table 9).

**Table 9.** Summary of two-way repeated-measures ANOVA results for P1

Effect	F	$df^{\mathrm{GG}}$	${\mathrm{df}}_{\mathrm{res}}^{\mathrm{GG}}$	p	$\eta_{p}^{2}$
Expression	0.06	1	17	0.802	0.004
Color	1.05	1.85	31.53	0.357	0.058
Expression × color	3.11	1.52	25.90	0.074	0.155

## Discussion

In this study, we used an oddball task to investigate whether the relationship between facial expression and facial color influences selective attention and recorded participants’ EEGs during the task. The results revealed that the P3 amplitude for red angry faces was greater than that for red neutral faces, suggesting that the EEG activity associated with the selective attention given to angry red faces was greater than that given to neutral red faces. P3 and selective attention are thought to be enhanced in response to threats (Öhman et al., 2001; Kessels et al., 2014). In addition, previous studies have reported that intensifying the redness of angry faces increases perceived emotional intensity, aggression, and threat (Thorstenson and Pazda, 2021; Thorstenson et al., 2022). Therefore, the increase in P3 amplitude for red angry faces might be attributed to the observer's strong sense of threat for that face stimulus. However, previous research has reported that the P3 amplitude is modulated due to semantic relevance (Kok, 2001). Since the task in this experiment involved counting the specified facial expressions, the increase in P3 amplitude for the red angry face might also be attributed to the semantic congruence between the emotion and the color as a contributing factor.

Moreover, facial expression and facial color had no main effect or interaction effect on the P1 amplitude, and facial expression had only a main effect on the N170 amplitude. In contrast, a main effect of facial expression and an interaction effect between facial expression and facial color were exerted for the P3 amplitude. These results indicate that the relationship between facial expression and facial color is represented by higher-order processing along the P1, N170, and P3 time axes. P1 amplitudes reflect the initial attentional processing of stimuli, and N170 amplitudes reflect differences in facial expression (Hillyard and Anllo-Vento, 1998; Hinojosa et al., 2015). In contrast, P3 amplitudes reflect higher-order cognitive processing, such as conscious attention (Polich, 2007). Hence, these findings suggest that enhancing responses resulting from the interaction between facial expressions and color are observed at later ERP stages than at early ERP stages associated with facial and facial color processing. As mentioned above, the P3 amplitude is modulated in association with stimuli (Kok, 2001). Additionally, involuntary differentiation processing for expressions is reported to occur later than N170 (Eimer and Holmes, 2002; Wronka and Walentowska, 2011). Therefore, the increased P3 amplitude for red angry faces might have been caused by the semantic processing of anger and red and later processing stages, such as the differentiation of facial expressions rather than simple facial color or expression processing.

In addition, the P3 amplitude, which reflects selective attention, is associated with memory. P3 is also an indicator of the degree of encoding and recall, and previous studies have suggested that a high P3 amplitude indicates the importance of encoding and the degree of successful recall (Karis et al., 1984; Fabiani et al., 1986; Polich, 2007). Emotionally relevant stimuli are known to be more strongly anchored in memory than are neutral stimuli, and the results of this study suggest that red angry faces have a greater influence on human memory (Dolcos and Cabeza, 2002). These results support previous studies that suggest that facial color memory for angry faces is biased toward more reddish and yellowish colors than that for actual facial color or neutral faces (Thorstenson et al., 2021; Hasegawa et al., 2024).

The results of this study revealed that the N170 amplitude depended on facial expression, with the N170 amplitudes for angry faces being larger than those for neutral faces. These results support findings from previous studies that emotional relatedness increases the N170 amplitude during facial processing (Hinojosa et al., 2015). However, the N170 amplitude did not differ among facial colors. The N170 amplitude reflects facial color processing, and the amplitude increases with respect to the unnaturalness of facial color (Minami et al., 2011; Nakajima et al., 2012). The stimuli used in this experiment had a color change of a*±12 units, which is considered not unnatural as a facial color. Thus, our results suggest that the a*±12 level of facial color change does not affect the N170 amplitude. This finding is also consistent with the results of Nakajima et al. (2012), where the N170 amplitude when the hue angle was changed in the red direction (−45° when the original color was 0°) did not differ from the N170 amplitude for the normal facial color (Nakajima et al., 2012).

The limitations of this study are as follows. First, the P3 amplitude is modulated not only by selective attention but also by factors such as memory performance and cognitive load (Polich, 2007; Minami et al., 2009). Consequently, the results of this study alone are insufficient to definitively establish whether the interaction between facial expression and color affects selective attention, and behavioral experiments showing increased selective attention to stimuli should be conducted.

Second, the individual characteristics of the participants were not researched in this experiment. The ability to detect faces, recognize or process facial expressions, and bias attention toward facial expressions such as angry faces varies depending on trait anxiety, autism spectrum disorder, or Moebius syndrome (Golarai et al., 2006; Surcinelli et al., 2006; Telzer et al., 2008; Tanaka and Sung, 2016; Quettier et al., 2023). Therefore, the individual characteristics of participants possibly may have affected differences in attention to facial expressions, and the differences in the magnitude of effects due to individual characteristics must be examined in the future.

Third, all participants in this experiment were Japanese, and the facial stimuli used were also Japanese models. Color preferences for emotion and facial color are known to vary across cultures, and the association between emotion and color is known to be developmentally variable (Boyatzis and Varghese, 1994; Han et al., 2018; Jonauskaite et al., 2020). Hence, it is appropriate to interpret the findings of this study as being based on phenomena observed under specific conditions, and their validity is limited within certain populations.

### Conclusion

We investigated whether ERP P3 varies with facial expression and facial color according to the hypothesis that humans bias more selective attention to red angry faces than to neutral facial expressions or original facial colors. The results revealed an interaction effect between facial expressions and facial colors on the P3, which was not observed in early responses such as P1 and N170, and the P3 amplitudes for red angry faces were greater than those for red neutral faces. These findings indicate that EEG activity is associated with selective attention to red angry faces rather than red neutral faces and suggest that the interaction between facial expression and color appears to enhance responses at a later cognitive processing stage than the enhancement associated with facial color or expression alone. Our findings support the idea that red increases or biases the response to anger from an EEG perspective.

## Data Availability

The analysis code and the data used for statistical analysis are available at https://osf.io/qvyfd/. However, the data are publicly available only as amplitude extraction data for each experimental condition of each participant, in accordance with regulations of the Ethics Committee for Human Research at Toyohashi University of Technology.

## Synthesis

Reviewing Editor: Niko Busch, Westfalische Wilhelms-Universitat Munster Decisions are customarily a result of the Reviewing Editor and the peer reviewers coming together and discussing their recommendations until a consensus is reached. When revisions are invited, a fact-based synthesis statement explaining their decision and outlining what is needed to prepare a revision will be listed below. The following reviewer(s) agreed to reveal their identity: Maria Olkkonen. Note: If this manuscript was transferred from JNeurosci and a decision was made to accept the manuscript without peer review, a brief statement to this effect will instead be what is listed below.

# synthesis

We have now received comments from two expert reviewers, and I would like to share their feedback and my own assessment with you. 

Both reviewers were generally positive about your work, highlighting the relevance of your research question and the quality of the experimental design. However, they provided several constructive suggestions for improving the manuscript, which I encourage you to address thoroughly. I recommend carefully addressing each of the comments.

The reviewers noted inconsistencies in the description of the rationale and motivational statements, and recommended clarifying the research questions and improving the justification for studying attention in this context. They also suggested expanding on the introduction of EEG components such as P1 and N170, similar to the treatment of the P3 component.

The reviewers raised concerns regarding the statistical analyses, including more comprehensive reporting of pairwise comparisons and clearer follow-up analysis for the interaction effects. Indeed, all statistical tests and their outcomes should be reported in the Results section.

There were also suggestions to consider additional behavioral experiments to strengthen the impact of the paper, although this is not mandatory. If these experiments are not added, please revise the discussion accordingly to temper conclusions about increased attention.

Additionally, both reviewers requested a more elaborate discussion. In simple terms, what have we learned about face perception in general, and specifically about the impact of face color?

I would like to add a few comments regarding the methods for EEG analysis:

188: "The remaining data were subsequently interpolated by the spherical spline interpolation method". Please clarify which data were interpolated. I assume this is referring to noisy channels-if so, how were these channels selected for interpolation?

199: ERP amplitudes were quantified in the time ranges of 80-120 ms, 150-200 ms, and 300-500 ms after the presentation of the standard and target stimuli. How were these time ranges determined? Additionally, how were the channels of interest identified?

204: Please clarify why one participant was excluded from the analysis.

Finally, I encourage you to make the data and code publicly available. I recommend mentioning the repository and including the corresponding link in the Methods section. See, for example, this paper (under CODE ACCESSIBILITY):

Microsaccades Track Location-Based Object Rehearsal in Visual Working MemoryEelke de Vries, Freek van EdeeNeuro, 4 January 2024, 11 (1) ENEURO.0276-23.2023; DOI: 10.1523/ENEURO.0276-23.2023

# Reviewer 1

The current manuscript describes a study whereby participants perform an oddball task requiring attention for faces having distinct facial expressions (neutral, angry) and facial color manipulations (neutral, redder, greener). EEG data are recorded during the task, with a focus on P3 amplitudes, and P1, N170 amplitudes. The results indicate varying differences among amplitudes attributable to facial expression, color, or their combinations. In my view, the experiments were well conducted and has the potential to contribute a novel perspective on perceptions of facial emotion with respect to nuanced attentional processes. Here, I describe several concerns and suggestions with the intention to improve the current manuscript.

-In both the Abstract and throughout the Introduction, there are several imprecise statements used to motivate the work. For example, the statements "...whether [facial expression and color] influence attention remains unclear", And "it remains unclear whether selective attention to faces is modulated by the relationships between facial expression and color" sound quite similar, but are proposing subtly different questions. I suggest more consistency in how these motivational statements (and others) are used throughout.

-Also related to motivating the current work, it appears that the initial justification given for studying attention in this context is simply because attention is "another cognitive function". Much more could be done to bolster the justification for why studying attention in this context is important, in the Introduction section.

-In framing the central research questions, two statements are given: "Therefore, the following questions arise: does a reddish angry face increase perceived threat and emotional intensity, and is the observer's attention more biased toward a reddish angry face than toward other faces?" The first statement is rather odd, given that perceived threat and emotional intensity are not studied in the current work at all.

-For audiences less familiar with interpreting specific EEG amplitudes (myself included), I found the section within the Introduction that describes P3 (what it is, how it is interpreted, etc.) quite helpful. However, given the additional analyses and findings concerning P1 and N170, I am unsure why these were not likewise introduced and explained. There is a brief section in the Discussion where these are mentioned, but perhaps this information should be elaborated on in the Introduction as well.

-I note for the Editor here that I do not have expertise in conducting EEG research, so I am unable to evaluate the EEG data processing, cleaning etc.

-In reporting the statistical comparisons for P3, A main effect of facial expression is reported (comparing neutral vs angry). An Expression*Color interaction is also reported, along with a follow-up comparison of red angry vs. red neutral. Given that this is a 2(expression)x3(color) interaction term, why is only one comparison reported for this interaction?

-Likewise, the statistical reporting in general is very minimal and could be improved - for example, there are several pairwise comparison and follow-up tests not reported which should be (even if found to be not statistically significant).

-The Discussion section focuses on interpreting the different amplitude effects independently, which is largely a reiteration of the Results sections. I had hoped to see a more wholistic interpretation, for example, what might the combination of amplitude effects observed in the current work mean regarding how humans attend to different emotional facial features, like expression and emotion?

-Also in the Discussion, about green-angry faces interpreted as incongruent and inducing cognitive load - While this is certainly plausible, it should be framed as speculation without empirical support in the current work. It could also be that green facial color tends to indicate aversive emotional states (e.g., disgust), and angry faces are also often confused with disgust, so there could be other perceptual interpretations and implications for the observed results that are not in line with the given interpretation. Please note that I am not suggesting to alter the authors' explanation (since mine also has no empirical basis from what is measured in the current work), I am only suggesting that such interpretations should be tempered in the current Discussion.

-The writing tense is sometimes inconsistent, for example using present tense to describe hypotheses, etc.

-Typo in figure 1B - "Angyr face"

# Reviewer 2

The authors use the P3 component of the EEG signal to investigate whether attention is drawn towards red angry faces more than neutral expressions or neutral-colored angry faces. I think the methods are sound and the manuscript is clearly written, but the impact of the paper could be larger if additional behavioral experiments showing increased attention to the stimuli were conducted. I also have a few concerns about the motivation and conclusions, listed below.

The research question in the introduction on line 90-91 reads: "does a reddish angry face increase perceived threat and emotional intensity, and is the

observer's attention more biased toward a reddish angry face than toward other faces?" It is not clear how the study addresses the first part of the question and this needs to be motivated or toned down.

In terms of the results, as the authors say, it is perhaps surprising that green angry faces also show a larger P3 component, although this effect is not statistically significant. If the authors still wish to discuss this trend, it introduces the question why there is an effect of green as well as red, and whether this somehow undermines the main conclusion about red angry faces.

Finally, the authors mention a couple of times that the effect of color red on attention is semantic - is this really a necessary conclusion? Could there not be perceptual-level interaction between facial expression and face color based on these data? I am not saying that it is so, but I think this needs some argumentation especially for people not familiar with EEG components.

A minor comment: the white point of the monitor (xy=[0.31, 0.35]) and the gray background chromaticity (xy=[0.3, 0.33]) don't match; is this intentional or a typo?

## Author Response

Response Letter We wish to thank the editor and reviewers once again. The insightful comments and suggestions have helped improve the manuscript considerably. We have modified the text and figures to better explain the contributions of our work.

A summary of the changes is as follows:

1. The overall text and figure were revised based on reviewers' comments.

2. Tables were added to report the results of the statistical tests.

3. No reviewer comments were noted, but corrections were made to the figures and statistical results. Details are given at the very end.

The detailed responses to the reviewers' comments are presented below.

------------------------------------------------------------------------------------------------------------------------- # Editor We have now received comments from two expert reviewers, and I would like to share their feedback and my own assessment with you.

Both reviewers were generally positive about your work, highlighting the relevance of your research question and the quality of the experimental design. However, they provided several constructive suggestions for improving the manuscript, which I encourage you to address thoroughly. I recommend carefully addressing each of the comments.

The reviewers noted inconsistencies in the description of the rationale and motivational statements, and recommended clarifying the research questions and improving the justification for studying attention in this context. They also suggested expanding on the introduction of EEG components such as P1 and N170, similar to the treatment of the P3 component.

The reviewers raised concerns regarding the statistical analyses, including more comprehensive reporting of pairwise comparisons and clearer follow-up analysis for the interaction effects. Indeed, all statistical tests and their outcomes should be reported in the Results section.

There were also suggestions to consider additional behavioral experiments to strengthen the impact of the paper, although this is not mandatory. If these experiments are not added, please revise the discussion accordingly to temper conclusions about increased attention.

Additionally, both reviewers requested a more elaborate discussion. In simple terms, what have we learned about face perception in general, and specifically about the impact of face color? We appreciate the time and effort you have invested in overseeing the review process and providing valuable insights that have undoubtedly raised the quality the manuscript. We have carefully checked and revised our entire manuscript based on your and the reviewer's comments.

I would like to add a few comments regarding the methods for EEG analysis:

1. 188: "The remaining data were subsequently interpolated by the spherical spline interpolation method". Please clarify which data were interpolated. I assume this is referring to noisy channels-if so, how were these channels selected for interpolation? Thank you for your feedback. As you noted, the noisy channels were interpolated with peripheral electrode data. We have revised the relevant sentence.

Page 7, Line 200: "The electrode data excluded by "clean_rawdata" were subsequently interpolated via the spherical spline interpolation method via the use of peripheral electrode data." 2. 199: ERP amplitudes were quantified in the time ranges of 80-120 ms, 150-200 ms, and 300-500 ms after the presentation of the standard and target stimuli. How were these time ranges determined? Additionally, how were the channels of interest identified? Thank you for your questions. We predefined the time windows and channels of interest during the experimental design phase based on previous research. We have added the following sentence to clarify why the time windows and channels were chosen.

Page 8. Line 213: "In this experiment, the time windows and channels used were predefined during the experimental design phase on basis of previous research (Che et al., 2024; Hinojosa et al., 2015; Polich, 2007)." 3. 204: Please clarify why one participant was excluded from the analysis.

Thank you for your comment. We have modified the text to clarify the reason for making this exclusion.

Page 8, Line 217: "Quartile range exclusion was subsequently performed for each condition to exclude outliers. Thus, the data of one participant with an outlier outside the interquartile range were excluded." 4. Finally, I encourage you to make the data and code publicly available. I recommend mentioning the repository and including the corresponding link in the Methods section. See, for example, this paper (under CODE ACCESSIBILITY):

Microsaccades Track Location-Based Object Rehearsal in Visual Working MemoryEelke de Vries, Freek van EdeeNeuro, 4 January 2024, 11 (1) ENEURO.0276-23.2023; DOI: 10.1523/ENEURO.0276-23.2023 Thank you for your suggestion. We have added the following sentences to the Data availability paragraph and published data at https://osf.io/qvyfd/.

Page 18, Line 392: "The analysis code and the data used for statistical analysis are available at https://osf.io/qvyfd/. However, the data are publicly available only as amplitude extraction data for each experimental condition of each participant, in accordance with regulations of the Ethics Committee for Human Research at Toyohashi University of Technology." ------------------------------------------------------------------------------------------------------------------------- # Reviewer 1 The current manuscript describes a study whereby participants perform an oddball task requiring attention for faces having distinct facial expressions (neutral, angry) and facial color manipulations (neutral, redder, greener). EEG data are recorded during the task, with a focus on P3 amplitudes, and P1, N170 amplitudes. The results indicate varying differences among amplitudes attributable to facial expression, color, or their combinations. In my view, the experiments were well conducted and has the potential to contribute a novel perspective on perceptions of facial emotion with respect to nuanced attentional processes. Here, I describe several concerns and suggestions with the intention to improve the current manuscript.

Thank you for your thoughtful review of our manuscript. We appreciate your positive feedback on the research question and the overall quality of the study. Your insights are valuable to us. On the basis of your comments and suggestions, the new manuscript has been improved. We believe that the revised manuscript satisfactorily addresses all your concerns. However, if any issues remain, please let us know. Thank you very much for your excellent review.

1. -In both the Abstract and throughout the Introduction, there are several imprecise statements used to motivate the work. For example, the statements "...whether [facial expression and color] influence attention remains unclear", And "it remains unclear whether selective attention to faces is modulated by the relationships between facial expression and color" sound quite similar, but are proposing subtly different questions. I suggest more consistency in how these motivational statements (and others) are used throughout.

Thank you for your suggestion. We have revised the text as appropriate to make it more consistent.

For instance, Page 2, Line 38: "However, whether these relationships influence selective attention and brain activity contributed to selective attention remains unclear." Page 2, Line 57: "Whether the relationships between facial expression and color influence selective attention and its brain activity remains unclear." 2. -Also related to motivating the current work, it appears that the initial justification given for studying attention in this context is simply because attention is "another cognitive function". Much more could be done to bolster the justification for why studying attention in this context is important, in the Introduction section.

Thank you for your advice. We have modified the relevant content of this manuscript.

Page 3. Line 84: "Red angry faces increase the perception of anger (Nakajima et al., 2017), whereas angry faces increase selective attention (Fox et al., 2002; Mogg et al., 2004; Öhman et al., 2001; van Honk et al., 2001). In the relationship between facial expression and selective attention, angry and fearful faces are known to bias visual attention, and it is believed that perceived threats may be among the factors that capture human attention (Fox et al., 2002; Mathews et al., 2003; Öhman et al., 2001). However, it is still unclear whether the relationship between facial expression and color affects selective attention and its brain activity, which are also modulated by facial expression. Therefore, we hypothesize that red angry faces, which are perceived as expressing more anger, also increase selective attention, and this hypothesis was tested via an approach based on electroencephalography (EEG)." 3. -In framing the central research questions, two statements are given: "Therefore, the following questions arise: does a reddish angry face increase perceived threat and emotional intensity, and is the observer's attention more biased toward a reddish angry face than toward other faces?" The first statement is rather odd, given that perceived threat and emotional intensity are not studied in the current work at all.

Thank you for your feedback. We have revised the relevant point in accordance with Comment 2.

4. -For audiences less familiar with interpreting specific EEG amplitudes (myself included), I found the section within the Introduction that describes P3 (what it is, how it is interpreted, etc.) quite helpful. However, given the additional analyses and findings concerning P1 and N170, I am unsure why these were not likewise introduced and explained. There is a brief section in the Discussion where these are mentioned, but perhaps this information should be elaborated on in the Introduction as well.

Thank you for your suggestion. We have added information about P1 and N170 to the Introduction section.

Page 4, Line 118: "P1 is an ERP component that occurs approximately 100 ms after a presented stimulus in the occipital area and reflects the initial visual processing (Colombatto and McCarthy, 2017; Hillyard and Anllo-Vento, 1998). Previous studies reported that P1 modulation was unaffected by facial expression factors (Schindler and Bublatzky, 2020). N170 is a negative ERP component that is observed approximately 170 ms after stimulus presentation in the left and right posterior temporal regions and is sensitive to facial stimuli (Bentin et al., 1996). N70 is modulated by facial expression and facial color (Hinojosa et al., 2015; Minami et al., 2011; Nakajima et al., 2012)." 5. -I note for the Editor here that I do not have expertise in conducting EEG research, so I am unable to evaluate the EEG data processing, cleaning etc.

Thank you for taking the time to review our manuscript.

6. -In reporting the statistical comparisons for P3, A main effect of facial expression is reported (comparing neutral vs angry). An Expression*Color interaction is also reported, along with a follow-up comparison of red angry vs. red neutral. Given that this is a 2(expression)x3(color) interaction term, why is only one comparison reported for this interaction? Thank you for your important question about improving our manuscript. We have abbreviated content to reduce the amount of text and the number of pages, but we have added the tables resulting from the statistical analysis.

7. -Likewise, the statistical reporting in general is very minimal and could be improved - for example, there are several pairwise comparison and follow-up tests not reported which should be (even if found to be not statistically significant).

Thank you for your important feedback about improving our manuscript. We have taken the same actions as previously stated (Comment 6).

8. -The Discussion section focuses on interpreting the different amplitude effects independently, which is largely a reiteration of the Results sections. I had hoped to see a more wholistic interpretation, for example, what might the combination of amplitude effects observed in the current work mean regarding how humans attend to different emotional facial features, like expression and emotion? Thank you for your great question. We believe that the amplitude differences observed in our experiment reflect differences in selective attention as modulated by the perceived threat level of the stimuli. We have modified the manuscript in the discussion section.

Page 15. Line 301: "P3 and selective attention are thought to be enhanced in response to threats (Kessels et al., 2014; Öhman et al., 2001). In addition, previous studies have reported that intensifying the redness of angry faces increases perceived emotional intensity, aggression, and threat (Thorstenson et al., 2021a; Thorstenson and Pazda, 2021). Therefore, the increase in P3 amplitude for red angry faces might be attributed to the observer's strong sense of threat for that face stimulus." 9. -Also in the Discussion, about green-angry faces interpreted as incongruent and inducing cognitive load - While this is certainly plausible, it should be framed as speculation without empirical support in the current work. It could also be that green facial color tends to indicate aversive emotional states (e.g., disgust), and angry faces are also often confused with disgust, so there could be other perceptual interpretations and implications for the observed results that are not in line with the given interpretation. Please note that I am not suggesting to alter the authors' explanation (since mine also has no empirical basis from what is measured in the current work), I am only suggesting that such interpretations should be tempered in the current Discussion.

Thank you for your helpful advice. As you mentioned, no significant difference was observed between angry faces and neutral faces in the green facial color condition, which was directly related to the experimental paradigm, and there was speculation in the discussion. We have removed the relevant discussion from our manuscript in response to your comment and Reviewer 2-Comment 2.

10. -The writing tense is sometimes inconsistent, for example using present tense to describe hypotheses, etc.

Thank you for your careful reading. We have adjusted the verb tense.

Page 4, Line 109: "Therefore, we hypothesized that when the relationship between anger and red causes strong selective attention, an interaction effect of facial expression and facial color is exerted on P3 rather than a color effect alone. For example, the P3 amplitude for red angry faces is larger than that for red neutral faces, and this trend is more pronounced than when angry faces with normal facial color are compared with neutral faces with normal facial color." 11. -Typo in figure 1B - "Angyr face" Thank you for carefully checking even the figure. We have revised the wording (Page 5).

------------------------------------------------------------------------------------------------------------------------- # Reviewer 2 The authors use the P3 component of the EEG signal to investigate whether attention is drawn towards red angry faces more than neutral expressions or neutral-colored angry faces. I think the methods are sound and the manuscript is clearly written, but the impact of the paper could be larger if additional behavioral experiments showing increased attention to the stimuli were conducted. I also have a few concerns about the motivation and conclusions, listed below.

Thank you for your thoughtful and positive feedback on our manuscript. We appreciate your encouraging remarks. Your insight into the behavioral experiments will serve as a valuable reference for our future research, and we have added this insight to the manuscript as a limitation of our study. We have revised the discussion to temper conclusions about increased selective attention, for example, "increased ERP associated with selective attention." Additionally, we have carefully considered your comments and suggestions and have made revisions accordingly. Please find the updated manuscript attached for your review.

1. The research question in the introduction on line 90-91 reads: "does a reddish angry face increase perceived threat and emotional intensity, and is the observer's attention more biased toward a reddish angry face than toward other faces?" It is not clear how the study addresses the first part of the question and this needs to be motivated or toned down.

Thank you for your feedback and suggestion. We have revised the relevant points and introduction to clarify the motivation and our approach in this study.

Page 3. Line 84: "Red angry faces increase the perception of anger (Nakajima et al., 2017), whereas angry faces increase selective attention (Öhman et al., 2001). In the relationship between facial expression and selective attention, angry and fearful faces are known to bias visual attention, and it is believed that perceived threats may be among the factors that capture human attention (Fox et al., 2002; Mathews et al., 2003; Öhman et al., 2001). However, it is still unclear whether the relationship between facial expression and color affects selective attention and its brain activity, which are also modulated by facial expression. Therefore, we hypothesize that red angry faces, which are perceived as expressing more anger, also increase selective attention, and this hypothesis was tested via an approach based on electroencephalography (EEG)." 2. In terms of the results, as the authors say, it is perhaps surprising that green angry faces also show a larger P3 component, although this effect is not statistically significant. If the authors still wish to discuss this trend, it introduces the question why there is an effect of green as well as red, and whether this somehow undermines the main conclusion about red angry faces.

Thank you for your helpful advice. As you mentioned, no significant difference was observed between angry faces and neutral faces in the green facial color condition, which was directly related to the experimental paradigm, and there was some speculation in the Discussion section. We have removed the relevant discussion from our manuscript.

3. Finally, the authors mention a couple of times that the effect of color red on attention is semantic - is this really a necessary conclusion? Could there not be perceptual-level interaction between facial expression and face color based on these data? I am not saying that it is so, but I think this needs some argumentation especially for people not familiar with EEG components.

Thank you for your excellent feedback and suggestion. As you noted, the possibility of a perception-level interaction between facial expressions and facial color can also be considered, and factors other than semantic processing might have influenced our results.

Therefore, we have revised the conclusion to "enhancing responses resulting from the interaction between facial expressions and color are observed at later ERP stages than at early ERP stages associated with facial and facial color processing." Additionally, we have added the following explanation to the Discussion section to support this conclusion.

Page 16, Line 318: "Hence, these findings suggest that enhancing responses resulting from the interaction between facial expressions and color are observed at later ERP stages than at early ERP stages associated with facial and facial color processing. As mentioned above, the P3 amplitude is modulated in association with stimuli (Kok, 2001). Additionally, involuntary differentiation processing for expressions is reported to occur later than N170 (Eimer and Holmes, 2002; Wronka and Walentowska, 2011). Therefore, the increased P3 amplitude for red angry faces might have been caused by the semantic processing of anger and red and later processing stages, such as the differentiation of facial expressions rather than simple facial color or expression processing." 4. A minor comment: the white point of the monitor (xy=[0.31, 0.35]) and the gray background chromaticity (xy=[0.3, 0.33]) don't match; is this intentional or a typo? Thank you for your comment. This mismatch is intentional. The white point described in the Apparatus paragraph is the measured value when the luminance of the monitor was set to a maximum (RGB = [255, 255, 255]), and the gray point described in the Stimuli paragraph has different RGB values (RGB = [138, 138, 138]) when output to the monitor. The gamma of the monitors differed slightly between the R, G, and B channels, causing differences in the two chromaticity points; however, since this difference was common throughout all the conditions, we do not believe it affected the results of this experiment. However, to avoid misunderstanding, the xy value of the gray background has been removed from the sentence (Page 5, Line 155).

---------------------------------------------------------------------------------------------------------------------- Finally, we have modified the notation text of the p value about P3 and the statistical symbols in Figure 4 as follows:

1. The p value for the comparison of angry red faces and neutral red faces for P3 was <0.05, but the correct notation was <0.01. Accordingly, the statistical symbol and its explanation at the relevant point of Figure 4 have been revised. Please check the Table 4 for the specific values. The conclusion was not changed.

Page 9. Line 253: "the P3 amplitude for red angry faces was greater than that for red neutral faces (t(17)=3.382,p<.01,〖Cohen〗^' s d=0.797)." 2. The statistical result symbols erroneously shown in Figure 4 have been removed. However, the conclusion was not changed.

Again, we hope these changes meet the expectations of the reviewers and the editorial team. We look forward to having our work reconsidered for publication in eNeuro.

Thank you for your time and consideration.

Sincerely, Yuya Hasegawa, Hideki Tamura, Shigeki Nakauchi, Tetsuto Minami
